# Exploring interspecific interaction variability in microbiota: A review

**DOI:** 10.1016/j.engmic.2024.100178

**Published:** 2024-11-09

**Authors:** Zhong Yu, Zhihao Gan, Ahmed Tawfik, Fangang Meng

**Affiliations:** a School of Environmental Science and Engineering, Sun Yat-sen University, Guangzhou 510006, China; b Guangdong Provincial Key Laboratory of Environmental Pollution Control and Remediation Technology, Sun Yat-sen University, Guangzhou 510275, China; c National Research Centre, Water Pollution Research Department, Dokki, Giza 12622, Egypt; d Department of Environmental Sciences, College of Life Sciences, Kuwait University, P.O. Box 5969, Safat 13060, Kuwait

**Keywords:** Microbial community, Interspecific interaction, Interaction variability, Variability mechanism, Interaction characterization

## Abstract

Interspecific interactions are an important component and a strong selective force in microbial communities. Over the past few decades, there has been a growing awareness of the variability in microbial interactions, and various studies are already unraveling the inner working dynamics in microbial communities. This has prompted scientists to develop novel techniques for characterizing the varying interspecific interactions among microbes. Here, we review the precise definitions of pairwise and high-order interactions, summarize the key concepts related to interaction variability, and discuss the strengths and weaknesses of emerging characterization techniques. Specifically, we found that most methods can accurately predict or provide direct information about microbial pairwise interactions. However, some of these methods inevitably mask the underlying high-order interactions in the microbial community. Making reasonable assumptions and choosing a characterization method to explore varying microbial interactions should allow us to better understand and engineer dynamic microbial systems.

## Graphical abstract

**Figure:**
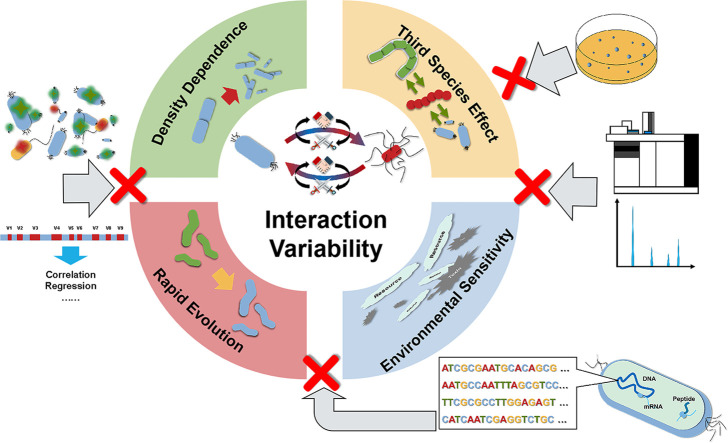

## 1 Introduction

Microbes widely exist in oceans, aquatic sediments, soil, animal guts, and engineering systems [1] and account for more than 50% of the total respiration on Earth [2]. It has long been acknowledged that these microorganisms usually live in communities [3,4], and strongly influence the reproduction and survival of their neighbors [5]. Biotic interactions comprise strong selective forces that determine the assembly and performance of microbial communities [6,7]. For instance, interspecific competition for available resources can shape the composition and mutually exclusive distribution of microbial communities [8,9]. Cooperative interactions have been implicated in community assembly and collective behavior because they can alter the nutritional quality of habitats and facilitate metabolite exchange among microbes [8,10]. In practice, microbial interactions vary under abiotic and biotic perturbations [10,11]. A previous study suggested that microbes could shift their interaction patterns after approximately 100 generations of co-adaptation [12]. Such interaction variability is expected to underlie the long maturation phase and poor stability of microbial communities [13], which in turn affects human health, environmental integrity, and biosystem stability. Therefore, capturing and understanding variations in microbial interactions will be pivotal in achieving efficient microbial engineering to solve the issues of infectious diseases, climate change, pollution control, and biosynthetic efficiency.

Exciting studies are now starting to uncover microbial interaction variability at various levels, such as the single-cell level [14], species-species networks [15], genetic manipulation [16], and environment-species association [17]. However, detecting the varying interactions caused by the interactive effects of different drivers is challenging. Several recent studies have proposed novel approaches to characterize various interactions based on changes in gene expression, metabolite accumulation, and cell concentrations over time. These methods will ultimately allow us to better understand the underlying mechanisms of microbial responses and variations in community performance under perturbations. The information derived using these methods should improve our ability to steer microbial communities toward a desired state through appropriate manipulation procedures [10,18].

Here, we summarize the current research investigating the variability in microbial interactions. In addition, we reviewed the applications of emerging methods that allow us to characterize various interactions, highlighting their strengths and weaknesses. Furthermore, we discuss how the ecological insights provided by these studies will shape future research and applications of microbes.

## 2 Definition of interspecific interactions

A rich body of research has described microbial interactions at different levels, but our understanding of interspecific interactions remains fragmented. What is lacking is an unified framework that defines the notion of microbial interactions for characterizing interaction variability in different systems [19].

### 2.1 Pairwise and high-order interaction

Pairwise interactions capture the relationships between two microbes, including cooperation, commensalism, predation, amensalism, and competition [20]. Specifically, cooperation and competition between two microbes represent positive and negative feedback, respectively [21]. In comparison, asymmetric interactions, such as commensalism or amensalism, indicate that one microbe can facilitate or inhibit its neighbor, but it is not affected by its neighbor. Predation is the exploitation of microbial prey by predators.

The language of graph is essential for the network-based modeling of microbiota systems [22] and can help capture the topological features of pairwise interactions. As an example, a graph G consists of a finite set of vertices $V=\{v_{0},...,\;v_{n}\}$ and a set of edges $E\subseteq \{\left\{v_{i},\;v_{j}\right\}\;|\;v_{i},\;v_{j}\in V\}$. The edge e can be either directed or undirected, representing the pairwise interaction between two microbes (i.e., vertices). In practice, the edge e is weighted to indicate the strength and sign of a given pairwise interaction. A recent study has suggested that interacting species in a community can have dual and immediate effects on each other (both beneficial *B* and harmful *H*) [23]. As such, there should be four unidirectional edges between two microbes (*i* and *j*) in a graph, and the net effect *M_ij_* exerted by *i* on *j* should be written as *M_ij_ = p_ij_B_ij_* - (1–*p_ij_*)*H*_ij_, where *p_ij_* represents the proportion of beneficial interactions. Moreover, different pairwise interactions within a community typically share the same node and form a multipartite interaction chain or loop. Thus, an upstream microbe in the interaction chain or loop can indirectly affect a downstream microbe by facilitating or inhibiting midstream microbes.

However, some microbes interact with each other in a higher-order manner and cannot be captured using pairwise interactions. Unlike interaction chains and loops, such high-order interactions indicate how a microbe modifies the pairwise interaction between two other microbes rather than one of the interacting microbes [24]. For example, in a co-culture of *Escherichia coli* and *Salmonella enterica*, phage addition to the T7 phage directly inhibited the sensitive *E. coli* rather than the non-host *S. enterica* [25]. However, this selective inhibition should be categorized as an interaction chain rather than a high-order interaction. In comparison, the antibiotic-degrading *Streptomyces* sp. can help sensitive *E. coli* flourish upon exposure to antibiotic-producing *Streptomyces* sp. [26]. Such interactions can be defined as high-order interactions because the antibiotic-degrading strain affects the pairwise interactions between its neighbors, not by facilitating or inhibiting one of them, but by degrading the chemical linkage between them (i.e., antibiotics). In this case, a hypergraph H is required to establish a high-order interaction network in a microbial community [27]. Compared to a graph, a hypergraph consists of a finite set of vertices $V=\{v_{0},...,\;v_{n}\}$ and a set of hyperedges E⊆P(V), where P(V) is the power set of V that consists of all subsets of V (e.g., $\left\{v_{1},v_{2}\right\}$, $\left\{v_{3},v_{7},v_{n}\right\}$, and so on) [22]. Simplicial complexes and simplexes are essential in high-order networks. A simplicial complex is a collection of simplices, and an m-simplex is a nonempty set of *m*+1 vertices from V, which is a generalization of a polytope in a given dimension [28]. For example, a 2-simplex [a,b,c] is a triangle in a 2-dimensional plane, which can be used to represent the joint interaction among microbes a, b, and c; a 3-simplex [a, b, c, d] is a tetrahedron in 3-dimensional space and represents the joint interaction of four microbes. A smaller simplex volume represents a closer connection between microbes [27]. Notably, the simplex [a, b, c] does not have an equal hyperedge {a, b, c}. If we use the simplex [a, b, c] to represent the interspecific interaction among a, b, and c, it requires all subfaces, including line segments (i.e., [a, b], [a, c], [b, c]) and points (i.e., [a], [b], and [c]). However, in practice, microbes a, b, and c may interact only within a group. For example, microbe c can only have a high-order effect on the interaction between a and b and cannot impact a or b by itself. Therefore, line segments [a, c] and [b, c] in the simplicial complex are redundant. In comparison, hyperedges such as {a, b, c} relax this constraint and eliminate redundant line segments, which is a more general representation of high-order interactions among a, b, and c [28]. Therefore, hypergraphs provide a better representation of higher-order networks in complex microbiota than in simplistic complexes.

### 2.2 Unit of positive and negative interactions

The signs and strengths of microbial interactions are determined by their consequences at different levels (such as species, populations, and individuals) [29]. Most studies define positive and negative interactions at the community population and species levels, that is, by comparing the biomass of monocultures of given microbes and their co-cultures [12,30]. An interaction that results in high biomass production (or loss) is regarded as strong facilitation (or inhibition). However, greedily optimizing the biomass production of a community may not be advantageous for the long-term fitness of a microbe and may even trigger early community extinction [31]. In fact, like any living system, individual microbes have a tendency toward selfishness, trying to maximize their benefits rather than that of the population [32,33]. The low immediate reward of species growth may help slow-growing microbes receive access to more resources [34], which guarantees a high future reward for the community (e.g., stability and biodiversity) [31]. Therefore, biases can be introduced if biomass variation is considered the gold standard for microbial interactions. Microbial interactions have different values and units in the same ecological context, which indicate outcomes at different levels of interest.

## 3 Factors modulating variations in microbial interactions

**Fig. 1:**
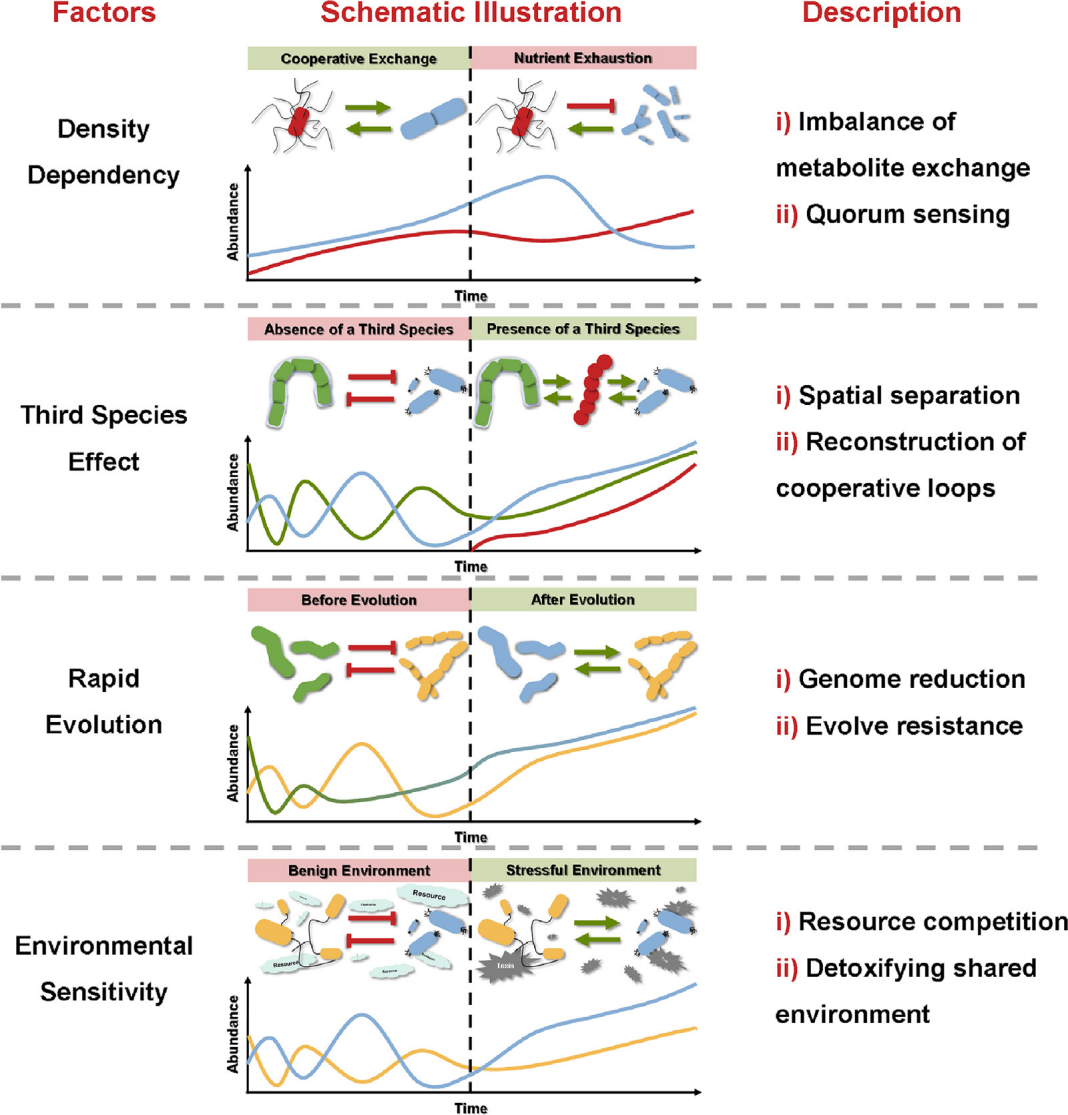
Effect of density dependency, third species effect, rapid evolution, and environmental sensitivity on microbial interactions.

Many factors have been proposed to regulate microbial interactions, including the metabolic state of the microbe, community structure, and the surrounding environment. Strong experimental evidence suggests that these factors can determine the interaction features (including topology, sign, and strength) of microbial communities in different ways. In this section, we describe key concepts by which these factors regulate the variability of interspecific interactions (Fig. 1 and Table 1).

**Table 1:** The modulating factors of microbial interaction variation

Modulating Factor	Mechanisms	Examples	References
Density dependence	● Imbalance of metabolite exchange	● *Bacillus megaterium* and *Ketogulonicigenium vulgare* can facilitate each other via metabolite exchange when *B. megaterium* is in low abundance. The increased abundance of *B. megaterium* will lead to the nutrient exhaustion of *K. vulgare*.	(Zhou et al. 2011)
	● Quorum sensing (QS)	● The higher cellular density increase the probability of recieving QS signals for microbes.	(Li and Tian 2012)
Third species effect	● Spatial seperation	● Sensitive *E. coli* strain can survive the antimicrobial pressures by the antibiotic-producing *Streptomyces* sp. in the presence of a antibiotic-degrading *Streptomyces* sp..	(Kelsic et al. 2015)
	● Reconstruction of cooperative loops	● *Streptomyces coelicolor* to produce at least 12 different desferrioxamines with acyl side chains in the presence of actinomycetes, which are not detected in *S. coelicolor* monoculture	(Traxler et al. 2013)
Rapid evolution	● Genome reduction	● *E. coli* and *Legionella pneumophila* can evolve amino acid auxotrophies and become dependent on the cross-feeding from community.	(D'Souza and Kost 2016, Price et al. 2014)
	● Evolve resistance	● *E. coli* can quickly evolve resistance against T4 phage and cooperate with other nonhost microbes in community.	(Fazzino et al. 2020)
Environmental sensitivity	● Resource competition	● Antibiotic production can benefit the *Saccharomyces cerevisiae* strain in the nutrient-rich environment with dispersal limitation but in nutrient-poor environment, the metabolic cost of toxin production outweighs its benefit.	(Wloch-Salamon et al. 2008)
	● Detoxifying shared environment	● The detoxification by *Agrobacterium tumefaciens* and *Comamonas testosteroni* can benefit *Microbacterium saperdae* and *Ochrobactrum anthropi* under nutrient-rich environment, but such benefit will vanish under nutrient-poor environment.	(Piccardi et al. 2019)

### 3.1 Density dependence

Growing evidence indicates that changes in the abundance of a single species can alter its fitness and population dynamics within a community [6,35]. For example, in a co-culture of *Bacillus megaterium* and *Ketogulonicigenium vulgare*, both mutualistic and antagonistic interactions are present [36]. When *B. megaterium* is present in low abundance, it can benefit from exogenous metabolites secreted by *K. vulgare*. In return, it releases erythrose, erythritol, and inositol to support the growth of *K. vulgare*. However, an increase in the abundance of *B. megaterium* leads to nutrient exhaustion in *K. vulgare; K. vulgare* will then induce the sporulation of *B. megaterium* to maximize its own benefit. In complex communities, interspecific interactions exhibit strong density dependence [37]. Variations in species abundance can potentially disrupt the coexistence of species if stabilizing processes (i.e., negative frequency dependence) are lacking in the local community [6]. In particular, a high population density is relatively unfavorable for cooperation in consuming public goods. Some microbes (referred to as “cooperators”) produce public goods that are costly to a focal cell but promote the survival and reproduction of neighboring cells (referred to as “cheaters”) [5]. According to the law of chemical equilibrium, recipient cheaters can accelerate the metabolism of cooperators by consuming the excreted metabolites [38]. Therefore, the combined metabolism of multiple species can fully utilize the capabilities of their environment [39]. Nonetheless, a higher density of cheating cells allows them to invest less in cooperation but gain a greater share of the public goods produced by cooperators [37]. Such asymmetric interactions eventually increase the fitness cost to the producing cells and even change positive interactions into negative ones [14,36]. However, an increase in cell density can be either favorable or unfavorable for cheating cells that use resources quickly but inefficiently, depending on their biological characteristics [37]. Regarding interspecific competition, a taxon with more individuals can obtain a survival advantage over chemical warfare across multiple taxonomic domains [40]. Therefore, a high cell density is normally required to neutralize the inhibitory effects of toxins or antibiotic producers in a community.

A typical mechanism associated with the density dependency of microbial interaction is the phenomenon of quorum sensing (QS) in microbes. QS is ubiquitous in bacterial communities [14], where bacterial cells communicate through QS molecules [41]. Some QS molecules, such as N-acyl-homoserine-lactone, can increase the microbial synthesis of extracellular polymeric substances (EPS) [42], which enhance the production of public goods in a community [43]. QS can mediate biofilm formation and protect microbes from the inhibitory effects of their competitors [43,44]. Specifically, QS increases the negative charge on the cell surface, facilitating bacterial interactions during biofilm formation [45]. Increased cell density favors the exchange of interspecific communication signals, promoting cooperative activities and physiological processes in communities [46].

### 3.2 Third species effect

A cross-feeding lifestyle makes the interaction between two microbes susceptible to perturbations by a third species in the community [25]. During microbial encounters, microbes elicit an appropriate cellular response to both self and non-self through direct contact or metabolite secretion [15,47]. Invasive microbes can occupy the niche between two native competitors, separating them into different spatial niches and neutralizing their chemical warfare [26,48]. This is supported by experimental evidence showing that antibiotic-degrading microbes attenuate the inhibitory effects of antibiotic producers on sensitive species [26]. For example, a sensitive *E. coli* strain is inhibited by antibiotic-producing *Streptomyces* sp., but can survive such antimicrobial pressures in the presence of antibiotic-degrading *Streptomyces* sp. [26]. Intriguingly, an experimental study suggested that negative pairwise interactions in co-culture do not become positive interactions upon the introduction of a third species [49]. However, the presence of a third species can stimulate the production of chemical compounds that are not produced when the native species grows alone [50]. For example, actinomycetes trigger *Streptomyces coelicolor* to produce at least 12 different desferrioxamines with acyl side chains that are not detected in *S. coelicolor* monocultures [50]. Microbes exchange these metabolites with specific partners and form close cooperative loops that indirectly benefit all species involved [9]. Such cooperative loops help microbes efficiently use limited resources [38], thereby developing a strategy for the coexistence of diverse niches [8,51].

In some cases, invasive microbes (e.g., phages) alter interspecific interactions by killing sensitive cells in communities and changing the species ratios [25,52]. These invaders can help resistant species flourish by killing their sensitive competitors and providing them with better access to limited resources [52,53]. Some resistant species can grow on cellular debris released by the lysis of their sensitive neighbors [25]. Nevertheless, it should be noted that the effects of a third species on the interaction between two microbes are generally species-specific. A closely related third species that shares similar ecological characteristics and functional traits with the native species is more likely to affect the local interaction [6,54]. Moreover, variations in a low-degree node can affect a system with a dense and homogeneous interaction network [18]. However, variations in single-network hubs have limited effects on sparse and heterogeneous networks [18].

### 3.3 Rapid evolution

Microbes constantly and rapidly alter their genetic material, contributing to their evolutionary success [55,56]. The mutation rate of microbes has been reported to range from 10^−10^ to 10^−9^ per nucleotide site per generation [[56], [57], [58]]. Moreover, plasmids can help microbes exchange genetic material and enable the transfer of plasmid accessory functions [59]. Within a microbial community, the collective microbiota genome can dramatically change the configuration of its components [60], ultimately affecting interspecific interactions.

Some microbes have evolved to have a compact genome that retains the necessary metabolic pathways [61]. As such, microbes lose most of their functional genes and increase their dependency on their co-occurring neighbors [62]. Experimental observations suggest that microbes such as *E. coli* can evolve amino acid auxotrophies in less than 2000 generations with an external supply of amino acids [63]. Similar phenomena have also been observed in other bacteria, such as *Legionella pneumophila*, which have evolved cysteine dependence [64]. Such emergent dependency has led to the transition of the microbial lifestyle from free-living to obligate symbiotic [61]. Conflicting reports have highlighted the evolution of cooperative exchanges as both beneficial and detrimental to microbial communities. More specifically, some studies report that microbes can evolve at molecular levels to establish specific partnerships in a community [38,65]. Such evolution is expected to help microbes exchange metabolites and use waste products from their neighbors efficiently, thus exerting a strong influence on productivity [65]. However, a previous study suggested that, owing to the spatial structure and social insulation, these cooperative exchanges in microbial communities are often limited and can be inefficient for group living [5]. Therefore, the productivity of a symbiont community is lower than that of an autonomous strain that makes everything it needs [5]. Moreover, some studies suggest that microbes may lose their capacity for DNA uptake and recombination as a consequence of genome reduction [66], leading to evolutionary dead ends [61].

Some microbes can evolve resistance and neutralize the inhibition imposed by their competitors. This may be the case in experiments involving interactions between antibiotic producers and sensitive cells or between a lytic bacteriophage and its host [25,67]. For example, upon T7 phage infection, phage resistance can quickly evolve in *E. coli*, increasing microbial yield [25]. During the evolutionary process, mutations allow some individuals within the population to attain alternative phenotypes; thus, intraspecies genomic diversity can be observed [68,69]. Phenotypic variability confers a selective advantage to these species [56], allowing them to better survive inhibition by competitors and resume their growth quickly. Moreover, plasmids can confer resistance to microbes and mediate metabolic exchanges among competing species, thus facilitating a beneficial relationship [59].

### 3.4 Environmental sensitivity

Interspecific interactions are sensitive to and adapt to changing environments through microbial phenotypic plasticity [70,71]. Unlike intraspecies genomic diversity, phenotypic plasticity is independent of genetics [68]. Thus, a genotype can exhibit different phenotypes depending on its response to different environments [70,72].

Environmental resources and stress are the dominant structural forces that affect biotic interactions. Microbes tend to facilitate each other in stressful environments but compete when resources are abundant [17,73]. Producing toxins that kill other organisms is an important way for microbes to gain and defend their niches when resources are abundant [40,74]. For example, antibiotic production can benefit *Saccharomyces cerevisiae* strains in nutrient-rich environments with limited dispersal [74]. However, in nutrient-poor environments, the metabolic cost of toxin production outweighs its benefit [74]. Moreover, it is difficult for microbes to maintain competitive phenotypes; hence, competition decreases over time [65]. Microbial interactions then become mostly positive, and microbes can use limited resources more efficiently [38]. Under highly toxic conditions, some species, like *Agrobacterium tumefaciens* and *Comamonas testosteroni*, must detoxify the environment for their own benefit, which also facilitate the neighboring microbes (like *Microbacterium saperdae* and *Ochrobactrum anthropi*) incapable of detoxification [17]. As environmental toxicity decreases, the benefit of detoxification is outweighed by the cost of detoxifying microbes. Instead, the competition between cheaters and detoxifying microbes for resources dominates. Hence, detoxifying microbes may no longer provide benefits to their neighbors in such scenarios [17,73]. The shift between cooperation and competition between two species is driven by selfish traits at the individual level [75]. Natural selection favors such selfishness, which can maximize the overall benefit to focal cells [76,77].

In addition, a structured environment plays an important role in regulating interspecific interactions [78]. In planktonic ecosystems, microbes share the same habitat and are prone to compete with each other [79]. Meanwhile, microbial interactions in a structured environment (e.g., a biofilm) tend to occur among cells in close proximity, owing to the diffusion-mediated exchange of molecules [14,80]. Microbial encounters can also potentially alter the production of microbial metabolites [47], thus affecting pairwise interactions. For example, two cross-feeding *E. coli* strains can increase the growth rates of their surrounding auxotrophic partners via the exchange of proline and tryptophan; however, such an interaction range (i.e., the distance between interacting microbes) is only on the order of a few cell lengths [80]. Both theoretical modeling and experimental evidence suggest that mutualism can be promoted by a structured environment within an intermediate interaction range; however, it is difficult for microbes to survive spatial expansion [81,82]. Meanwhile, solid or semi-solid structures can neutralize interspecific competition by allowing competing microbes to coexist in a spatially “frozen” pattern [40]. This effect was previously described in a study of localized interactions between toxins-producing bacteria [40].

### 3.5 Interactive effects

In practice, multiple factors can affect interspecific interaction [83]. For example, the abundance-adaptation hypothesis suggests that a high cell density allows a taxon to undergo more evolutionary activities to adapt to a new environment [84]. Therefore, these species are expected to experience changing interactions with their neighbors. Moreover, it has been found that phylogenetically close strains tend to cooperate, while phylogenetically distant ones are likely to compete with each other [77]. However, high relatedness and low niche overlap may negate the effects of genotypic dissimilarity on microbial interaction patterns [77]. The interaction between distinct genotypes may also change from negative to positive under the effects of environmental stochasticity and the presence of immigrant species [85]. In addition, microbes are more inclined to evolve auxotrophy and become dependent on their neighbors when living primarily within or on a larger organism than free-living microbes in water, soil, or sediment [86].

Hamilton's rule from social evolution theory indicates that the interspecific interaction pattern is governed by the balance between its benefits and costs to the microbes being observed [14]. In other words, a cooperative or competitive trait is favored if

br−c>0

where *c* is the fitness cost of an action for microbes, *b* is the fitness benefit provided by their neighbors after an action, and *r* is the relatedness between the focal cells and their neighbors. These parameter values may vary with cell density, the presence of a third species, microbial genomic evolution, and environmental variation, resulting in changes in the interaction pattern. For example, the cost of plasmid carriage for a microbe is canceled out in the presence of competitors [87]. Therefore, identifying the combined and separate effects of these factors on interspecific interaction patterns will allow us to better understand and regulate dynamic microbial networks.

**Fig. 2:**
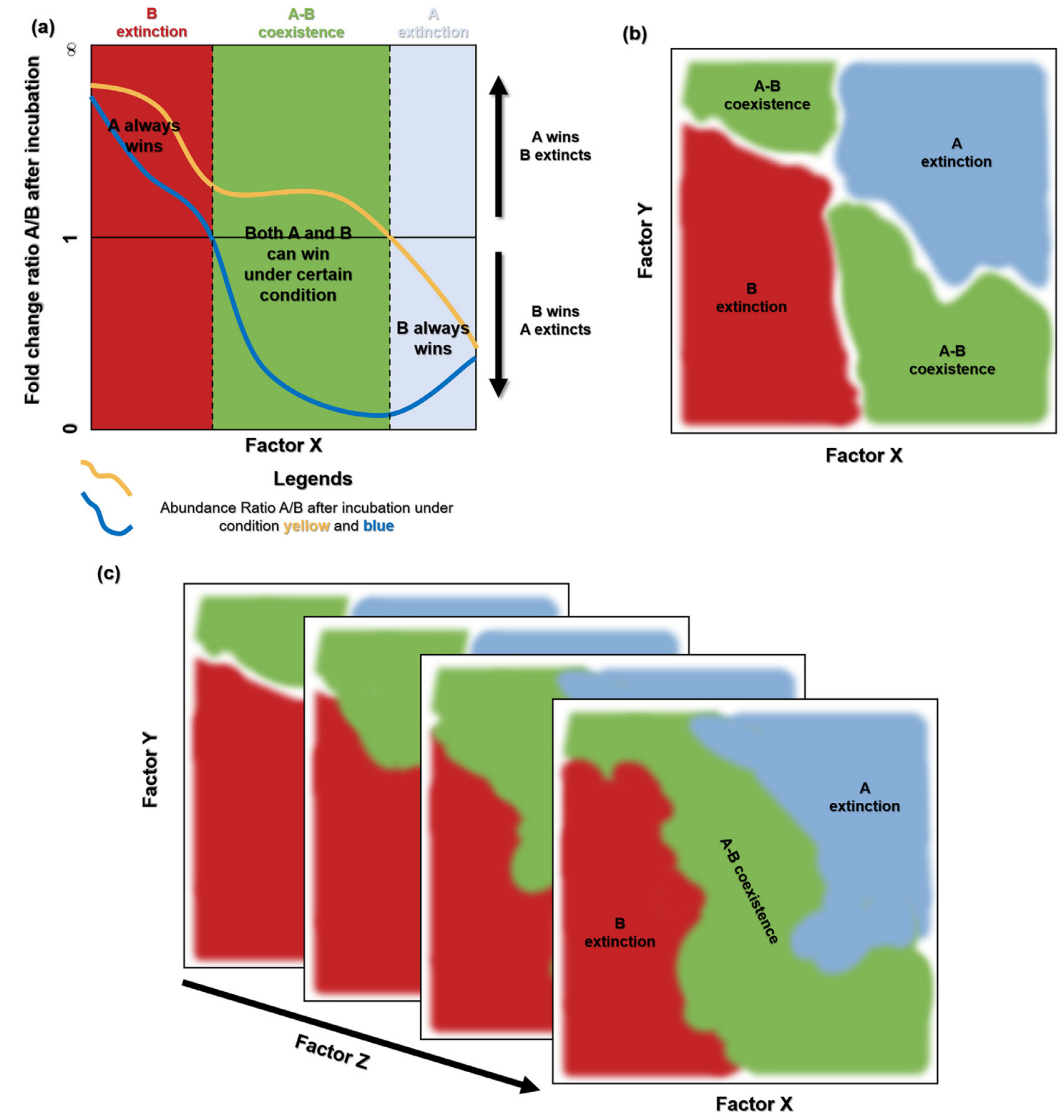
Schematic illustration of the effect of multiple factors on microbial interactions. (a) Consider a co-culture system with two strains, A and B. The MKS model developed by Gude et al. can estimate the fold change ratio A/B after different incubation conditions (yellow and blue lines) as a function of factor X. With hierarchy inversion, A is found to outcompete B when A/B is > 1, whereas B outcompeted A when A/B is < 1. When there is no hierarchy inversion, A consistently outcompetes B, resulting in the extinction of B (red region), or B always wins, which leads to the extinction of A (blue region). A and B are driven to an equilibrium ratio (i.e., coexistence) if they can “win” under specific conditions (green region). (b) The effect of dual factors (X and Y) on the interaction between A and B according to (a). X and Y can determine the interaction between A and B in an interactive manner. At low X and Y, B will be outcompeted (red region); at high X and Y, A will be outcompeted (blue region); otherwise, A and B can coexist (green region). (c) The interaction between A and B displays a complex pattern under the interactive effect of three factors (X, Y, and Z).

There have been increasing efforts to develop ecological frameworks to estimate the contribution of multiple factors to microbial interaction variability [78,88]. For example, Gude et al. proposed the Monod–Keller–Segel (MKS) model [88] based on the Keller–Segel [89] and Monod equations [90]. The MKS model can accurately simulate the inversion of the competition hierarchy for co-culture under changing conditions such as growth rate, migration speed, chemotactic sensitivity coefficient, colony radius, nutrient consumption, and nutrient diffusion coefficients (Fig. 2). They estimated the interactive effects of phenotypic mutations, initial community structure, and environmental variation on the coexistence of bacteria in a co-culture. Intriguingly, both simulation and experimental evidence support the idea that bacterial coexistence can break down when a factor crosses a specific threshold. Such abrupt shifts in contrasting states (i.e., tipping points) have been widely observed in ecosystems [70]. However, the framework developed by Gude et al. requires a justified assumption regarding the governing equations of community dynamics [88], which fails to consider the effect of a third species. However, despite these limitations, this framework advances our understanding of the multifactor-based mechanisms of microbial interaction variability.

## 4 Characterizing microbial interactions

**Fig. 3:**
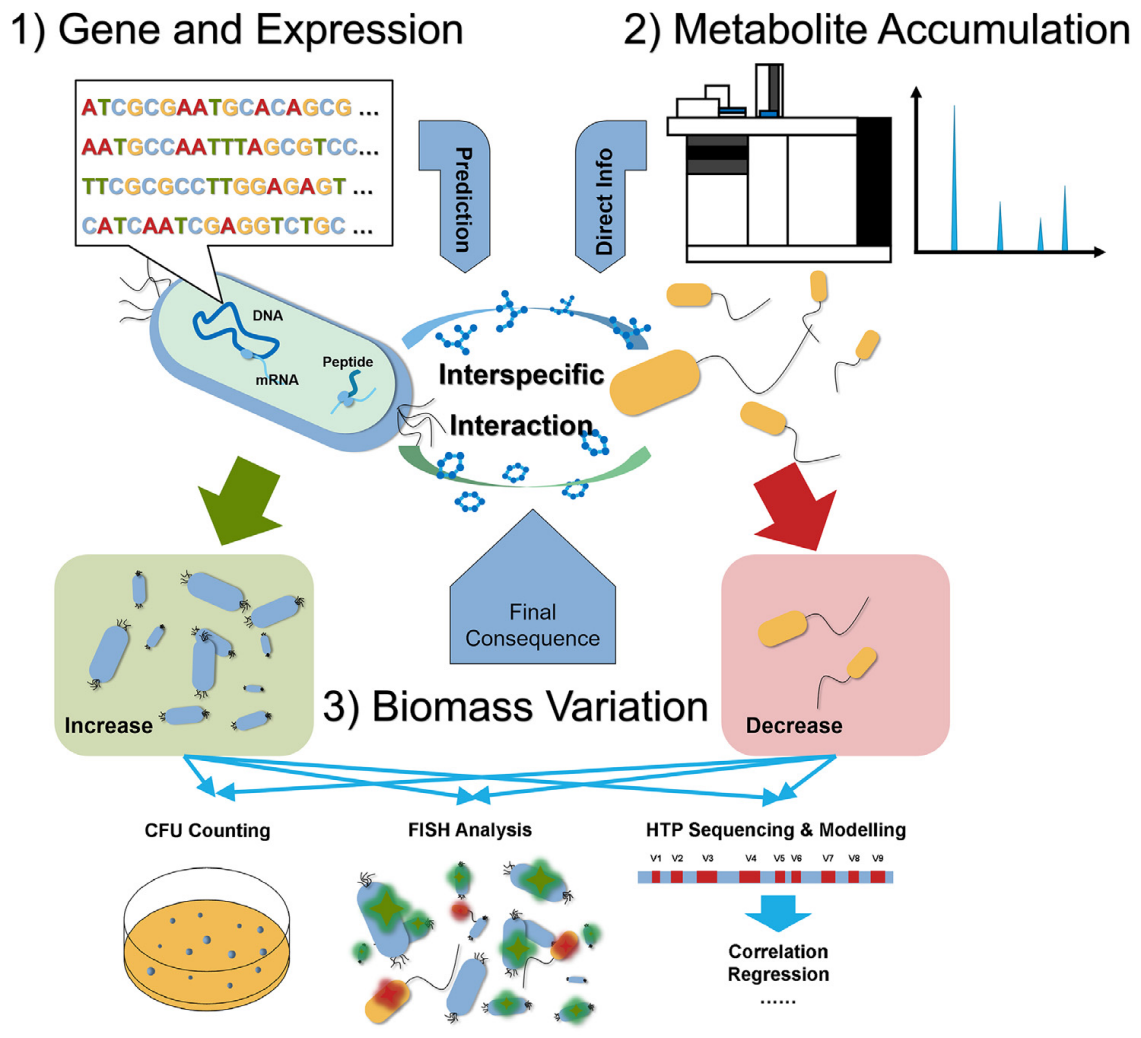
Characterization of microbial interaction based on changes in gene expression, metabolite accumulation, and biomass variation over time.

Variations in microbial interactions can be characterized through gene expression, metabolite accumulation, and biomass variations. However, given the complex mechanisms of variability in microbial interactions, these characterization methods have strengths and weaknesses (Fig. 3 and Table 2).

**Table 2:** Comparison among different characterization methods for microbial interaction variation

Strategy			Advantages	Limitations	Application Scope	Selected References
**Prediction by gene expression**	**Genomics-based**	● Taxonomy-based prediction by PIDA or PICURSt	● Cheap data collection	● Neglect the importance of rapid evolution	Potential Pairwise interaction and high-order interaction	(Bjorbækmo et al. 2020, Langille et al. 2013)
		● Genome-based metabolic modelling	● Visualize the metabolic exchange pattern among microbes	● Limited knowledge of the gene function		(Harcombe et al. 2014, Orth et al. 2010)
			● Disentangle the role of rapid evolution	● Issues of gene silencing		
	**Transcriptome and proteome-based**	● Quantification of functional gene transcription and protein	● Provide information regarding metabolic activity of microbes	● Unknown functions of mRNA and protein		(Wisnoski et al. 2020, Jonkers et al. 2012, Hettich et al. 2012)
				● Integrity issue for mRNA extraction		
**Metabolite accumulation analysis**	**Quantification of extracellular metabolite**		● Capture specific microbial interaction	● Prior knowledge required for the metabolite	Pairwise interaction; high-order interaction	(Oliveira et al. 2014, Zhou et al. 2011, Yeon et al. 2009)
	**Metabolomic similarity**		● Capture the global picture of microbial metabolism	● Identities of considerable compounds remain unknown	Pairwise interaction	(Sedio et al. 2018, Watrous et al. 2012)
**Monitoring biomass variation**	**Similarity-based**	● Pearson & Spearman Correlation	● Wide application	● Issues of delayed association	Pairwise interaction	(Faust and Raes 2012, Kodera et al. 2022)
			● Easy interpretation			
		● Local Similarity	● Unveil the delayed association	● Compositional effect caused by relative abundance		(Ruan et al. 2006)
		● SparCC	● Resolve the issues of compositional effect	● Third species effect not considered		(Friedman and Alm 2012)
	**Regression-based**	● Generalized Lotka-Volterra Model, etc.	● Infer multi-species interaction	● Correct assumption and extensive data required	Pairwise interaction; high-order interaction (requiring additional assumption for model)	(Hoffmann et al. 2007, Mounier et al. 2008, Sidhom and Galla 2020, Taylor 1988)
				● Unmeasured cofounding		
				● Overfitting issue		
		● Dynamic Linear Model	● No assumption about community dynamics equation	● Underfit when ecosystem changes rapidly		(Deyle et al. 2016, Lamon III et al. 1998)
		● Empirical Dynamic Modelling	● Address the state dependence	● Curse of dimensionality		(Chang et al. 2021, Deyle et al. 2016, Yu et al. 2020)

### 4.1 Interaction prediction based on gene expression

Advances in sequencing technology have improved our understanding of microbial taxonomy, genomic structure, genetic mutations, gene expression, and metabolic networks [91]. Information obtained through these technologies can facilitate in-depth analyses and predictions of microbial interactions and their variability. For example, Bjorbækmo et al. registered 2422 interspecific interactions from 531 publications and built the *Protist Interaction DAtabase* (PIDA) [92]. PIDA can identify the ecological relationships (e.g., parasitism, predation, and symbiosis) between two microbes based on the taxonomic classification of their sequences at the genus and species levels. Nonetheless, most studies often neglect the interactive effects of microbial genotype/phenotype, community structure, and environmental factors [83]. Therefore, it is difficult to accurately identify interspecific microbial interactions based on phylogenetic classification and PIDA information [13]. Numerous studies have reported that genomic sequences can be utilized to compare genetic maps between microbes and reconstruct their metabolic networks [13,93,94], shedding light on the prediction of varying microbial interactions. For example, PICRUSt (*Phylogenetic Investigation of Communities by Reconstruction of Unobserved STates*) is a useful algorithm for predicting the functional capabilities of microbes using the 16S marker gene sequences of bacteria and archaea [94]. Based on this, a small-scale animal study reported an increase in carbohydrate metabolism along the intestine of grass carp, which underlies the increasing microbial cooperation from the fore to the hind intestines [95]. Nevertheless, it is noteworthy that such predictive functional profiling by marker genes neglects the importance of the rapid evolution of microorganisms.

In comparison, reconstructing metabolic networks from genomic data can help acquire more accurate information regarding microbial interaction patterns [9,96]. Some modeling frameworks (e.g., *Computation of Microbial Ecosystems in Time and Space*, COMETS) can compute the spatiotemporal dynamics of interacting microbes using genome-scale metabolic models [97]. These prediction results can be used to explore and visualize metabolic exchanges among microbes [98]. In these studies, metabolite flow and colony growth were predicted using *Dynamical Flux Balance Analysis* (dFBA) and biochemical network stoichiometry [99]. More recently, a novel reinforcement learning algorithm was developed to unveil the shift in microbial metabolic strategies by treating metabolism as a decision-making process [31]. Therefore, we expect that genome-scale metabolic modeling will deepen our understanding of varying microbial interactions under different cultivation conditions, genetic evolution, and community structures.

With the development of metagenomic binning [100], individual draft genomes of uncultivated microbes can be extracted from metagenomic data and used to reconstruct a metabolic network of the community. To date, metagenomic binning has been widely applied in studies on microbial interactions in natural and engineered systems (e.g., activated sludge [101], anaerobic digestors [102], hot springs [103], and acid mine drainage [103]). In addition, population-scale genome sequencing can detect structural variants in microbial populations [104], which may disentangle the role of rapid evolution in the variability of microbial interactions. Furthermore, a genetic map of the microbial genome can help explain the mechanisms underlying microbial interaction patterns. For example, single-cell genomic analysis was used to examine 127 genomes of uncultivated SUP05 bacteria and document bacteria-virus relationships [93]. This study found that some viruses can insert foreign genes into the host genome, thus reprogramming energy metabolism and enhancing the population of their host [93]. Some existing approaches, such as comparative genomics, could be used to quantify horizontal gene transfer [105], providing evidence regarding the effects of modified metabolic networks on microbial interaction variability.

However, a major bottleneck in genomics-based approaches is our limited knowledge of gene function [106]. For example, although the genome sequence of the type strain *E. coli* has been published in its entirety, ∼1/3 of its protein-encoding genes remain functionally unknown [107]. Moreover, predicting microbial auxotrophy from genome sequences remains premature, and contradictory results have been reported regarding the distribution of bacterial amino acid auxotrophies [86,108]. Recent decades have witnessed growing advances in the field of protein function prediction (e.g., AlphaFold) [109,110]. However, such efforts have been dampened because the expression of some genes strongly depends on environmental parameters [94,111]. Individual studies have reported that many genes in various species (e.g., *E. coli* and fungi) are repressed under laboratory growth conditions [112,113]. Therefore, genome-based analyses may fail to reveal the varying patterns of microbial interactions due to gene silencing.

Transcriptomics and proteomics are powerful tools that provide information about the metabolic activity of microbes. Sequencing analysis of 16S rRNA genes and their transcripts has led to the discovery of the limited role of terrestrial bacterial immigration in active aquatic communities, although terrestrial immigration could promote the local diversity of recipient communities[114]. Exploring the up- and downregulation of functional genes (e.g., those involved in amino acid synthesis) can provide functional profiles in great detail, which helps interpret the dynamics of metabolic exchange among microbes [115]. Nonetheless, RNA extraction is labor-intensive [116], and it is difficult to meet the requirements for preserving mRNA integrity [117]. Moreover, it is still cost-intensive to gather sufficient transcriptomic profiles, thus limiting their application in the detailed monitoring of microbial interactions. Recently, the development of single-cell transcriptomic analysis has allowed us to access expression profiles that are several-fold larger than bulk transcriptomic profiles, which capture subtle changes in interaction dynamics. [118,119]. However, gene transcription does not necessarily represent gene translation. For example, in a co-culture of *Ustilago maydis* and *Fusarium verticillioides*, the accumulation of fusarin C was not observed despite the upregulation of genes from the fusarin C cluster [115]. Despite the possibility of extraction errors, this observation highlights the shortcomings of the transcriptomics-based approach to some extent. Similarly, metaproteomics investigates various microbial interactions at the protein level [120]. Unfortunately, such methods require prior knowledge of the protein function [8], and thus far, many proteins have no assigned functions [115]. Therefore, a combination of protein function prediction and multi-omics approaches is required to explore the underlying mechanisms of microbial interaction variability.

### 4.2 Direct information via metabolite accumulation analysis

Microbes interact with each other through metabolite exchange. On the one hand, considerable microorganisms reported so far live in an auxotrophic lifestyle [121]; that is, they cannot synthesize essential nutrients and rely on cooperators in cross-feeding communities [122]. On the other hand, distributed metabolism is common for the biodegradation of complex organic compounds by the microbial community, where one microbe uses the by-product of its neighbors as a resource [123]. Numerous studies have uncovered the profound impacts of such metabolic exchange between microbes on the structure and functionality of microbial systems [79,124,125]. As such, detailed information on microbial interactions should be encoded at some level during metabolite accumulation in a local community [60]. Compared to genome- and proteome-level analyses, metabolomes can provide more intuitive and precise information regarding microbial interactions [36,106].

Characterizing extracellular metabolites (i.e., the metabolic footprint) enables the analysis of metabolic crosstalk among interacting microbes [126]. In this scenario, we need to select appropriate biomarkers to characterize metabolic exchange between microbes. For example, amino acids play key roles in primary and secondary metabolism [36]; however, most microorganisms fail to produce all standard amino acids *de novo* and derive most of them from the environment [122,127]. Therefore, amino acids serve as a currency for cooperative exchange among microbes [5], making them ideal biomarkers for elucidating microbial metabolic states [36]. For example, amino acid content has been used to reveal metabolic cooperation in co-cultures of *B. megaterium* and *K. vulgare* [36]. Specifically, a low intracellular amino acid content in *K. vulgare* indicates its deficiency in primary metabolism, which leads to a requirement for the metabolites released by *B. megaterium*. Moreover, certain secondary metabolites can also act as biomarkers of microbial interactions [113]. Vitamin B_12_ and other cobamides are required by microorganisms in all domains, but only a limited number of species can synthesize these nutrients [128]. It is expected that the extracellular cobamide level can provide clues to the metabolic exchanges between cobamide-producing cooperators and cheaters. Notably, the exchange of amino acids may not be sufficient to maintain the growth of microorganisms, given that half of the dry weight of microbial biomass is protein [86]. Microbes require vitamins at low concentrations; thus, the exchange of vitamins, rather than amino acids, should play a more important role in the interaction among microbes.

Investigating metabolite degradation is an alternative approach for characterizing microbial interactions at the metabolite level. For example, bile acid biotransformation in the gastrointestinal tract is primarily performed by *Clostridiales* [129,130]. Detecting residual bile acids in the environment could reveal the cooperative and inhibitory effects of other microbial community members on *Clostridiales*. Moreover, signaling molecules, antibiotics, and toxins have also been widely used for characterizing microbial interactions. Signaling molecules (e.g., N-acyl homoserine lactone) in a local community help reveal cooperative group behaviors among microbes (e.g., biofilm formation) [131], and the content of lytic debris in the environment could also aid in determining the strength of interspecific competition [15]. In addition, studies focusing on gastrointestinal microbiota have demonstrated the inhibitory effect of secondary bile acid deoxycholate on *Clostridium difficile*, suggesting its potential role in characterizing the competition between *C. difficile* and other microbes [132,133]. Interestingly, anthracyclines released by *Streptomyces* have been found to inhibit *Acinetobacter* [134,135], and such metabolites may provide crucial information regarding specific microbial interactions. In recent years, machine learning has gained popularity in selecting phenotypic biomarkers [136]. This method is expected to provide additional biomarkers and novel guidelines for future studies, opening a new avenue for investigating microbial interactions.

Additionally, the overall profile of the microbial metabolome can play a role in the characterization of interspecific interactions. In one study, the concept of metabolomic similarity was employed to predict interspecific interaction [137]. It acknowledged that chemically similar species tend to compete with each other, while those with greater differences in secondary metabolites are more likely to coexist [137]. To date, the identities of a considerable number of compounds discovered via mass spectrometry (MS) remain unknown [50]. Several approaches have been proposed to maximize the chemical and biological interpretability of MS data [138,139]. For example, the biological relevance of unknown compounds can be estimated using molecular networks and similarity scores between molecules and their identified analogs [138]. Such information would allow us to better explore the association between structural and compositional similarities among compounds and microbial interactions.

Monitoring metabolite levels in living microbes can provide important information for tracking interspecific interactions in a community [138]. Several experimental techniques have been developed to perform sensitive metabolic profiling of living microbes, such as *nanospray Desorption Electrospray Ionization Mass Spectrometry* (nanoDESI MS). NanoDESI MS enables *in situ* surface liquid extraction of microbial metabolites and feeds the compounds into a mass spectrometer via electrospray. Compared to the traditional MS approach, nanoDESI MS makes capturing metabolite production in living microbes possible without destructive extraction. Furthermore, *Matrix-Assisted Laser Desorption/Ionization Imaging Mass Spectrometry* (MALDI-IMS) can be used to visualize the spatiotemporal distribution of metabolites secreted by different microbes [47]. These methods are expected to reveal the varying metabolic profiles of interacting microbes; however, they are labor-intensive and require expensive equipment. Moreover, previous studies have shown that microbes cannot synthesize some metabolites when grown alone [50,113]. Therefore, a combination of cultivation-based methods and MS analysis may fail to quantify metabolic interactions in large microbial networks [140]. Finally, microbes often share or produce the same metabolites, making it hard to attribute a given metabolite to a particular member in a complex community [8,141,142]. Consequently, the MS approach cannot distinguish the effects of individual taxa on the variation in community metabolic networks, limiting its application in the study of complex systems. Recent developments in microfluidic approaches have allowed us to monitor the interactions between degraders and cross-feeders in a community at the single-cell level [123]. Soon, the combination of real-time MS, single-cell measurements, and stable isotope probing of novel biomarkers is expected to provide more insights into the metabolite exchange flux among microbes.

### 4.3 Estimation of interaction patterns by monitoring biomass variation

Biomass variations are expected to be a direct consequence of interspecific interactions. Therefore, comparing microbial productivity in monocultures and mixed cultures should provide direct evidence of microbial interactions [4,49,143]. Some cultivation-based studies have elucidated microbial interaction patterns based on the biofilm biomass of a co-culture [144,145]. Briefly, the pairwise microbial interactions were grouped into synergistic induction, induction, unresolved, reduction, and antagonistic reduction based on the differences in the biomass of the co-culture and monocultures. More specifically, synergistic induction indicates that the co-culture produces more biomass than the sum of those of monocultures; induction indicates a greater biomass production of co-culture than the maximum biomass production of monoculture. Similarly, antagonistic reduction and reduction represent the less biomass production of co-culture than the average and minimum of biomass productions of monocultures, respectively. This methodology should reveal changes in microbial interactions under different conditions. Similarly, a study of microbe-phage interactions compared the growth rates of different community members to investigate the effects of temperature and mutation on interspecific interaction variation [146]. However, such an approach requires measuring growth rates in mixed cultures and can only be applied to species with distinct phenotypes. In addition, the aforementioned approaches can only detect interactions between culturable species; 99% of microorganisms remain unculturable using traditional approaches [147]. Furthermore, the effect of high-order interactions on pairwise interactions is neglected by these methods [49], failing to accurately estimate the true interactions in a microbial community [13]. Notably, such methodologies are experimentally extensive, limiting their application in exploring large microbial networks.

In recent decades, the development of molecular biotechnology has advanced our understanding of the diversity and relative abundance of microbial communities, enabling us to understand interspecific interactions [92,148]. For example, a fluorescence *in situ* hybridization-based survey quantified the relative abundances of anammox bacteria and heterotrophs in anammox biofilms using confocal laser scanning microscopy [149]. Correspondingly, the varying autotroph-heterotroph interactions were also investigated by comparing the bacterial growth rates under different nitrogen loading rates and biofilm thicknesses. Additionally, high-throughput sequencing and clustering algorithms have enabled the quantification of the relative abundances of all microbes in natural and engineered systems [148]. Numerous studies have also investigated microbial interactions using mathematical models and the relative abundances of operational taxonomic units (OTUs) [3,[150], [151], [152]]. These methodologies fall into two categories (similarity-based and regression-based) according to their mathematical models (Table 1) [3,152].

Similarity-based methods quantify microbial interactions simply by the distribution similarity of interacting microbes and the significance of the similarity score, including the Pearson correlation coefficient and Spearman's rank correlation coefficient [3,153]. A positive similarity score indicates co-occurrence between interacting microbes, while a negative one represents co-exclusion [7]. Some studies successfully employed similarity-based methods to explore variations in microbial interactions under changing conditions [[154], [155], [156], [157]]. One of the challenges in resolving microbial networks is the appropriate definition of the correlation threshold [158,159]. Integrating random matrix theory into ecological network analysis can improve the characterization of network topological properties [160]. However, it should be noted that coexistence or co-exclusion does not equate to positive or negative interactions among microbes. These terms describe the state of the community structure rather than interspecific interactions in the community. Coexisting or co-excluding microbes do not necessarily interact with each other owing to spatial segregation [14,80]. Only in the equilibrium community are co-occurrence and co-exclusion equivalent to interspecific cooperation and competition, respectively. Owing to the metabolite diffusion limit [14,80], microbes often display delayed associations with each other [161]. Accordingly, time-lagging interactions can direct microbial co-occurrence and co-exclusion during community succession [162]. Thus, it is important to reveal time-lagged interspecific interactions. Ruan et al. proposed the use of local similarities to identify delayed associations between microbes [161]. They imposed a constraint on the delay between two matching time series and quantified the interaction strength by calculating the maximum similarity score of all the subsequences within the time delay. Moreover, quantifying microbial interactions by local similarity can resolve the issue of delayed association; however, it has some important limitations. Usually, genomic survey data can only represent the relative rather than absolute abundance of community members [163]. Therefore, a simple definition of interspecific cooperation and competition based on the similarity of relative microbial abundances could lead to severely biased and false results. For example, in a co-culture system, two microbes can enhance each other's absolute biomass yield through mutual cooperation. However, the changes in their relative abundances might exhibit opposite trends owing to different growth rates, which will be regarded as competition using similarity-based methods. Therefore, a novel algorithm called *Sparse Correlations for Compositional data* (SparCC) was developed to resolve the issues of compositional effects in network analysis [163]. Briefly, it quantifies the interspecific interaction by comparing the variances of the log-ratio transformation and log-transformed basis abundance of two interacting OTUs. Quantification of interactions based on the ratio of the relative abundance of microbes is expected to represent biotic effects on their absolute abundance. However, such pairwise correlation analyses fail to consider the effects of a third species [10,163].

In comparison, regression-based methods can infer multi-species interactions via multiple regressions [3]. These methods assume that the dynamics of microbial populations could be approximated using a set of equations, e.g., the generalized Lotka–Volterra (gLV) model [[164], [165], [166], [167]]. The gLV model plays a central role in elucidating the community ecology and could describe a microbial system at the population level [168,169]. Regression methods such as the Bayesian inference algorithm can be used to extract sets of interaction constants for the gLV model that reproduce observed patterns in the real microbiome (e.g., the empirical probability distribution of species abundance and pairwise correlation) [168,170]. However, these methods require prior knowledge of the correct form of the model [171,172], and different model assumptions could lead to controversial conclusions. For example, conflicting reports highlight high-order interactions as both important and neglectable factors in the microbiota assembly [21,173]. Specifically, Venturelli et al. [21] assumed that per capita growth rates are determined by monospecies growth and pairwise interactions between microbes, as shown in the following equation:

$$\frac{1}{x_{i}}\cdot \frac{dx}{dt}=\mu_{i}+\sum_{j}^{n}\alpha_{ij}x_{j}$$

where *x_i_* indicates the absolute abundance of species *i*, which is estimated as the product of its relative abundance and the OD_600_ value of the community. *μ_i_* and *α_ij_* represent the monospecies growth rate of species *i* and the effect of species *j* on itself, respectively. Notably, they concluded that high-order interactions play a minor role in community assembly because they can make accurate predictions for most species with information on monospecies growth and pairwise interactions alone, without defining high-order interactions in their model.

In comparison, Bairey et al. [173] assumed that per capita growth rates are determined not only by pairwise interactions but also by multipartite interactions, as shown in the following equations:

$$\frac{1}{x_{i}}\cdot \frac{dx}{dt}=f_{i}\left(\vec{x}\right)-\sum_{j}^{n}f_{j}\left(\vec{x}\right)x_{j}$$

$$f_{i}\left(\vec{x}\right)=-x_{i}+\sum_{j}^{n}\alpha_{ij}x_{j}+\sum_{k}^{n}\sum_{j}^{n}b_{ijk}x_{j}x_{k}+...$$

where *b_ijk_* is the joint effect of microbes *j* and *k* on *i*. They concluded that high-order interactions shape the diversity of complex microbiota because their model could reveal the effect of a third species on monospecies growth and pairwise interactions. To avoid such contradictory conclusions, the goodness of fit of the models under different assumptions needs to be evaluated, and conclusions should be drawn from the model that best reproduces the abundance pattern. Moreover, some parameters should be experimentally measured before model construction, such as the growth rate, microbial expansion rate, and diffusion coefficient of the nutrient, etc. [88,172]. Extensive data are required to obtain these parameters and construct a model [174]. However, the parameter values of certain phenotypes may vary with genome evolution, community structure, and the environment [172]. Moreover, the unmeasured confounding and overfitting issues of a parametric model can undermine model prediction performance [3,175]. Together, these limitations hinder the application of regression-based methods for the study of various microbial interactions in practical systems [3].

In recent years, non-parametric models have received significant attention in studies on interspecific interactions in macroecosystems [176,177]. The non-parametric model makes no assumptions regarding the governing equations of community dynamics. It calculates the interaction strength using multivariate linear regression with the minimal assumption that the change in a species at a given time point is a function of the current ecosystem state [178,179]. Deyle et al. inferred varying interspecific interactions in a five-species food chain model using a *Dynamic Linear Model* (DLM) [176]. DLM can reconstruct the interaction strength in a near-equilibrium linear system but may underfit when the ecosystem changes rapidly [176,180]. Accordingly, they proposed *Empirical Dynamic Modelling* (EDM) based on ecological state-space reconstruction. The EDM builds an attractor manifold from the abundance time series of a community by projecting the community's dynamic trajectory onto a multidimensional state-space. Therein, the geometric properties (i.e., partial derivatives) of the manifold represent the varying interspecific interactions under the interactive effects of genotypes, phenotypes, community structure, and environment. A locally weighted multivariate linear regression scheme (S-map) was developed to infer the matrices of the partial derivatives of the attractor manifold. The S-map successfully resolved the changing patterns of interaction strengths and signs among calanoid copepods, nanoflagellates, picocyanobacteria, and rotifers in the Baltic Sea. Based on this, further work improved the accuracy of S-map inference by imposing an elastic net regularization function [181]. Despite the curse of dimensionality, the S-map algorithm has greatly improved our ability to explore various microbial interactions in complex systems [10,140,182].

### 4.4 The application scopes of different methods

Given the strengths and weaknesses of these methods, selecting the optimal method is critical for accurately characterizing pairwise and high-order interactions. Interaction prediction based on gene expression is not ideal for determining microbial interactions. This is because it focuses on the functioning of microbial cells rather than that of the entire community. Therefore, this method can only provide information on potential pairwise and high-order interactions. For example, the upregulation of genes involved in amino acid synthesis may imply a pairwise interaction between the producing microbes and their auxotrophic neighbors; the expression of detoxification genes may indicate the high-order effect of a third microbe on the inhibitory interaction between toxin-producing and sensitive microbes. In comparison, quantifying extracellular metabolites in the microbial community should provide more direct information on pairwise and high-order interactions. In other words, the accumulation of amino acids and degradation of toxins may represent pairwise and high-order interactions, respectively. Notably, the microbial metabolomic similarity between two microbes may not represent the topological features of high-order interactions with multiple microbes. Regarding biomass variation-based methods, we suggest that similarity-based methods can only be used for pairwise interaction characterization because they fail to capture the effect of a microbe on other pairwise interactions. In comparison, regression-based methods can be used to characterize pairwise interactions and have the potential for quantifying high-order interactions. Specifically, most existing regression-based methods (e.g., the gLV model) make assumptions without considering high-order interactions. Introducing an additional assumption regarding higher-order interactions into the model should enable us to capture higher-order interactions and better reproduce the observed variation in the microbial community.

Most microbes live in an open community system whose properties are largely determined by biotic and abiotic factors [183]. From an engineering perspective, it is critical to determine the effective input/output (e.g., substrate ratio/biomass synthesis rate) of the target microbiota system and make reasonable assumptions about the linkage between pairwise/high-order interactions and the system input/output. We need to clarify whether the microbial behavior of interest affects the interacting microbes or their linkages. In this case, combining different characterizations, rather than using only one method, is required to better capture variations in both pairwise and high-order interactions in the microbial community. This should guide us in designing a better control strategy to maintain the stability and enhance the productivity of the microbiota.

## 5 Concluding remarks

Microbes affect their neighbors in communities in a highly nonlinear and evolving manner. A rich body of work has shed new light on the factors that modulate the variability of microbial interactions. Novel techniques have enabled us to track varying interspecific interactions based on genome sequences, gene expression patterns, metabolite accumulation, and biomass variations in the interacting microbes. However, many technical issues remain regarding the accuracy of the measurements and the biological meaning of the results. Hence, it is of the utmost importance to determine the level at which microbial interactions are defined and how such knowledge serves the purpose of a study. Recent studies have generated massive amounts of multidimensional data on microbial interactions (e.g., metabolomic profiles and species abundance data). Carefully choosing the appropriate characterization methods will offer new insights into how the dynamic complexity of microbial systems affects human health, global warming, agriculture, and bioengineering. Such an in-depth understanding of microbial interaction variation should improve our skills in achieving efficient control over microbial systems and harness the true metabolic potential of microbes.

## Declaration of AI in Scientific Writing

No generative artificial intelligence (AI) and AI-assisted technologies were used in the writing process.

## CRediT authorship contribution statement

**Zhong Yu:** Writing – review & editing, Writing – original draft, Visualization, Investigation, Funding acquisition, Conceptualization. **Zhihao Gan:** Writing – original draft, Investigation. **Ahmed Tawfik:** Writing – review & editing, Funding acquisition. **Fangang Meng:** Writing – review & editing, Supervision, Funding acquisition, Conceptualization.

## Declaration of Competing Interest

The authors declare that they have no known competing financial interests or personal relationships that could have appeared to influence the work reported in his paper.
